# AI-Based Prediction of Gene Expression in Single-Cell and Multiscale Genomics and Transcriptomics

**DOI:** 10.3390/ijms27020801

**Published:** 2026-01-13

**Authors:** Ema Andreea Pălăștea, Irina-Mihaela Matache, Eugen Radu, Octavian Henegariu, Octavian Bucur

**Affiliations:** 1 Genomics Research and Development Institute, Bucharest 030167, Romania; ema-andreea.palastea2022@stud.umfcd.ro; 2 Faculty of Medicine, Carol Davila University of Medicine and Pharmacy, Bucharest 030167, Romania; irina.m.matache@gmail.com (I.-M.M.); eugen.radu@umfcd.ro (E.R.); 3 Emergency University Hospital, Bucharest 050098, Romania; 4 Department of Neurosurgery, Yale School of Medicine, New Haven, CT 06520, USA; 5 Viron Molecular Medicine Institute, Boston, MA 02108, USA

**Keywords:** machine learning, AI-based prediction, single-cell omics, quantum computing, deep learning, spatial transcriptomics, gene expression

## Abstract

Omics research is changing the way medicine develops new strategies for diagnosis, prevention, and treatment. With the surge of advanced machine learning models tailored for omicss analysis, recent research has shown improved results and pushed the progress towards personalized medicine. The dissection of multiple layers of genetic information has provided new insights into precision medicine, at the same time raising issues related to data abundance. Studies focusing on single-cell scale have upgraded the knowledge about gene expression, revealing the heterogeneity that governs the functioning of multicellular organisms. The amount of information gathered through such sequencing techniques often exceeds the human capacity for analysis. Understanding the underlying network of gene expression regulation requires advanced computational tools that can deal with the complex analytical data provided. The recent emergence of artificial intelligence-based frameworks, together with advances in quantum algorithms, has the potential to enhance multiomicsc analyses, increasing the efficiency and reliability of the gene expression profile prediction. The development of more accurate computational models will significantly reduce the error rates in interpreting large datasets. By making analytical workflows faster and more precise, these innovations make it easier to integrate and interrogate multi-omics data at scale. Deep learning (DL) networks perform well in terms of recognizing complex patterns and modeling non-linear relationships that enable the inference of gene expression profiles. Applications range from direct prediction of DNA sequence-informed predictive modeling to transcriptomic and epigenetic analysis. Quantum computing, particularly through quantum machine learning methods, is being explored as a complementary approach for predictive modeling, with potential applications to complex gene interactions in increasingly large and high-dimensional biological datasets. Together, these tools are reshaping the study of complex biological data, while ongoing innovation in this field is driving progress towards personalized medicine. Overall, the combination of high-resolution omics and advanced computational tools marks an important shift toward more precise and data-driven clinical decision-making.

## 1. Introduction

The process of gene expression is determined by the dynamic relationship between the cell and various intrinsic and extrinsic factors. The emergence of new technologies might help with deciphering the underlying mechanisms that direct a cell towards changing its state [1]. Understanding this process requires both high-resolution experimental techniques and computational tools capable of capturing the complexity of cellular diversity. Single-cell sequencing plays an important role in the research of gene expression [2], as it enables the analysis of genomic, epigenomic, and transcriptomic heterogeneity in cellular populations and the tracking of changes over time. Single-cell genome sequencing reveals genetic variability, compared to bulk sequencing, which only offers an average gene expression profile in populations of cells [3]. Bulk sequencing remains a cost-effective alternative, but it masks particular and rare patterns. The function of each cell is dictated by a regulatory network which comprises transcription factors (TFs) and regulatory sequences, which makes the approach of single-cell multi-omics critical for unveiling the biological contribution of every cell. The analysis of the transcriptome, the proteome, and the genome is employed in identifying the potential trajectory of a cell. Single-cell studies can be performed either one at a time or in a massive parallel fashion. Nonetheless, they have improved the way in which we understand the biological networks created in heterogeneous tissues [4]. Building on this expanding resolution of cellular states, it becomes important to clarify how these technologies connect the gap between genetic variation and functional consequences.

While genome-wide association studies (GWASs) managed to identify thousands of variants associated with the risk of genetically inherited traits and diseases, it did not reveal the mechanisms underlying these links [5]. Consequently, integrating transcriptomic and epigenomic modalities has emerged as a powerful strategy to explain how genetic variants exert regulatory effects.

Transcriptomics profiling can be combined with multimodal epigenetic analysis techniques such as single-cell chromatin overall omics-scale landscape sequencing—scCOOL-seq [4]; nucleosome occupancy and methylome sequencing (cNOME-seq) [6]; single-cell chromatin accessibility and transcriptome sequencing (scCAT-seq) [7]; and combined assay for transposase-accessible chromatin using sequencing and RNA (scATAC-RNA-seq) [8]. These methods, crossing multiple epigenetic layers, facilitate the mapping of accessible chromatin regions in single cells, another powerful approach for dissecting tissue heterogeneity [4].

Although scRNA-seq [9] (single-cell transcriptomics) and other single-cell techniques have become indispensable tools in multiple research areas, they have limitations (see Benefits and Limitations). One would be the risk of being presented only with a partial image of the complexity of the gene regulation process [4]. Nevertheless, its limitations arise from the lack of spatial context. To combat these issues, spatial transcriptomics (ST) technologies were introduced. This concept relies on retaining spatial information that highly contributes to gene expression analysis and prediction [9].

Dynamic cellular processes do not depend on the expression of a single gene but are determined by the temporal expression and function of multiple genes. This hints at the importance of the spatial interaction between different sites of the genome, the interaction with other cells, and with the surrounding environment. Even though single-cell omics and spatial genomics give us an unprecedented view on gene programs and cell states, these methods require the alteration or lysis of the cells (to obtain single cells) and prevent us from directly observing the dynamics of molecular profiles in living cells. Certain pseudo-time algorithms, such as Monocle2 [10] or Waddington-OT [11], can deduce dynamic processes from static snapshots of molecular profiles. Pseudo-time inferring represents the progression of single cells along a biological process, based on gene expression profiles, in the conditions of lacking explicit time points [12]. These methods rely on assumptions about RNA kinetics that might be violated when the sampling timescale does not align with the pace of biological changes. Technologies like Live-seq [13,14] provide real-time sampling, but with a low throughput.

The complex datasets have catalyzed the adaptation of artificial intelligence (AI) for suiting data interpretation [15]. A subset of AI, deep learning (DL), leverages artificial neural networks to extract patterns from data, their architecture being inspired by the biological model of animal brains. Each layer serves as a non-linear transformation function of its input, processing the result from the previous layer as its input and generating progressively complex representations as the number of layers increases. The training of DL models is performed by them fitting themselves on the training data. DL frameworks perform tasks with a deep and hierarchical architecture, making them suitable for biological applications. These features encouraged scientists to build DL models performing various predictive tasks, ranging from predictions regarding crop yields [16] to frameworks that estimate mortality rates [17] or assess risk stratification among patients with a certain condition, such as COVID-19 [18]. The similarity between biological sequences and natural languages enables DL architectures to process omics datasets [19], thus increasing their applications designed to predict regulatory interactions, model transcriptional regulation, or identify non-coding variant effects.

The training of neural networks to recognize and predict gene expression patterns represent an important upgrade towards precision and preventive medicine. From a clinical point of view, such models could perform early diagnosis, patient clustering, and treatment design [20]. Minor genetic variability can be incriminated for decreased response to treatment and AI can help identify such variants and find an alternative that suits the patient’s phenotype.

Nonetheless, machine learning (ML) and deep learning (DL) models encounter significant challenges in predicting mammalian transcriptional regulation due to the complex sequence logic and the need to analyze interactions over large genomic sequences [21].

The complexity of this type of analysis originates from the multiple variables that must be taken into account when inferring different predictions. mRNA vs. protein abundance relationship studies reveal interesting correlations [18,22,23], while mapping quantitative trait loci [24] allowed the switch from bulk to single-cell focus. The AI revolution brought interest in developing models that work with existing datasets and improve the quality of the gene expression profiles; DL platforms were created for understanding transcriptional regulation, the impact of noncoding variants, and many other directions of investigation [25]. As an example, DeepBind [26] is able to predict transcription factor-binding sites (TFBSs).

This review aims to summarize the recent advances and applications of AI algorithms in the study of gene expression, especially discussing the ability to predict expression patterns, highlighting the importance of single-cell omics in the development of this research area. This paper also tackles the topic of promising clinical applications that can result from gathering data through these innovative technologies; a brief description of the possible improvement that can be brought by quantum computing in this field is included as well.

## 2. Single-Cell Sequencing Principles and Protocols

Single-cell omics upgraded the resolution at which scientists look at tissue heterogeneity [27]. Compared to bulk sequencing, despite being more statistically sensitive and harder to analyze, it also reveals a more realistic and detailed version of tissue complexity [28]. There is a continuous quest for computational methods to link cell heterogeneity at gene expression level to phenotype [27]. Single-cell sequencing brought new perspectives not only in the diagnostic and identification of possible gene variants but also suggested new applications in drug discovery and development [29]. To understand how these advantages are realized in practice, it is necessary to examine the experimental workflows and technological variants that underlie single-cell assays.

Since the introduction of single-cell technology, various approaches have been applied in order to capture cells and to amplify RNA, each having advantages and disadvantages [30]. Provided that a single-cell technique by itself might only offer an incomplete frame of the complexity of the gene regulation process, the introduction of the multi-omics approaches certainly enhances the quality of the determinations [4].

While single-cell sequencing methods can be applied at multiple levels and spots, one of the most used technologies is the single-cell RNA sequencing (scRNA-seq), which captures the transcriptome for assessing gene expression levels.

The main steps in scRNA-seq involve single-cell isolation and capture, cell lysis, reverse transcription, cDNA amplification, and library preparation. Isolation and capture steps aim to yield high-quality individual cells from tissues, forming a basis for precise genetic and molecular analyses. ScRNA-seq preserves the individuality of each cell, empowering the study of heterogeneous populations such as tumors, unlike bulk sequencing, which offers an average signal from a large number of cells.

There are different methods of isolating and capturing cells, depending on the organisms, cell properties, or tissues [31]. Common strategies include isolating whole cells, nuclei, organelles, or using specific marker proteins to separate particular cell types. The most widely used methods are fluorescence-activated cell sorting (FACS), magnetic-activated cell-sorting (MACS), microfluidic systems, and laser microdissection. Each single cell is finally captured in an isolated reaction chamber where all transcripts from this single cell are uniquely barcoded after being converted into cDNA molecules.

Gradually, it has been shown that the dissociation process might induce the expression of stress genes, revealing an artificial alteration of the transcriptional process. Experiments demonstrated that protease dissociation at 37 °C triggers the expression of stress genes [30], whereas performing dissociation at 4 °C minimizes the artifact. As an alternative to the scRNA-seq for single-cell sequencing, single-nucleus RNA sequencing (snRNA-seq) can be used. This method avoids the need for intact cells, as it only targets the mRNAs from the cells’ nuclei. It can be applied to frozen or otherwise difficult tissue samples, besides minimalizing the artificial transcriptional stress response [32]. snRNA-seq reduces the artifacts induced by dissociation, has broad applicability, and the isolation process is easier, but it lacks information on mRNA processing and RNA stability.

Following RNA extraction and reverse transcription, cDNA amplification is performed using either polymerase chain reaction (PCR) [33,34,35,36,37,38,39] or in vitro transcription (IVT) [40,41,42,43]. Both methods can lead to amplification biases; therefore, the introduction of unique molecular identifiers (UMIs) that are used to barcode each mRNA molecule during reverse transcription marked a major improvement [44]. The listed sequencing protocols use adapted UMIs [37,38,45].

The next step involves sequencing barcoded cDNA libraries using high throughput platforms. In DNA nanoballs, DNA fragments are processed to generate a 3′ adenine overhang. DNA fragments are ligated with dTTP-tailed adapters, circularized and amplified to generate dense libraries. The final product of this protocol is a single-strand circular DNA library [1].

The isolation and capture strategies divide scRNA-seq techniques into two main approaches, the plate-/microfluidic-based methods and droplet-based methods. The plate- and microfluidic-based methods are similar and often limited to ~50 to ~500 cells per analysis. Plate-based systems use fluorescence-activated cell sorting, while microfluidic-based ones are automated platforms, such as Fluidigm C1, which use parallel microfluidic channels. Fluidigm C1 uses valve-based isolation, which controls the fluidic circuit; this valve system is placed under the connection site of the nanoliter chambers [46]. These methods generally present high sensitivity, quantifying up to ~10,000 genes per cell. Meanwhile, droplet-based methods barcode single cells and use UMIs to tag each transcript in individual oil droplets [2]. This increases the throughput to up to 10,000 cells per run. However, it only detects 1000–3000 genes per cell, due to technical limitations. To put it briefly, the first method performs a more sensitive gene quantification but can cope with a smaller number of cells in an analysis, while the latter extracts less genetic information from each cell, but provides a transcriptional landscape from a larger population of cells. Droplet microfluidic protocols have also been developed, such as spinDrop [47], which mixes fluorescence-activated droplet sorting (FADS) for obtaining the probe with picoinjection. Regardless of the chosen platform, rigorous quality control is indispensable to ensure that downstream analyses reflect true biological signal rather than technical artifacts.

Choosing a scRNA-seq method requires considering a few factors, such as the expected number of cells, cell size, and acceptable technical biases. Quality control (QC) is crucial and is divided into cell QC and gene QC. For cell QC, barcodes that might not belong to intact individual cells should be eliminated. These altered cells are filtered out by analyzing UMI counts, the number of expressed genes, the total detected counts, and the proportion of mitochondrial RNA fractions. For high throughput scRNA-seq, doublets or multiplets must also be excluded—these are cells that present very high counts and a large number of expressed genes and occur in the case of two or more cells that are accidentally directioned in the same droplet or well [26,48]. Since none of the sequencing methods alone can tell the differences between doublets and true single cells, computational tools, such as scMODD (a model-driven doublet detector in scRNA-seq data) [49], scrublet [50], and scds [51], have been developed to infer and remove these artifacts [30].

## 3. Machine Learning and Deep Learning Models Used for Gene Expression Levels Prediction

### 3.1. Machine Learning and Deep Learning Architectures in Omics Research

AI systems are designed to perform tasks in the same way a human being would. These models imitate human cognition and are constructed in such a manner that they develop learning, reasoning, and perception like a human. This concept empowered the scientific world to develop AI-based systems that are more and more complex and that can analyze large amounts of data, recognize patterns, and make predictions based on the learnt and extracted information. Furthermore, ML and DL frameworks can learn from previous mistakes and adapt afterward even without being programmed to do so. Machine learning is a subset of AI that focuses on building algorithms that enable computers to learn from data and make predictions. DL is a subset of ML which uses artificial neural networks to learn from data. The similarity of artificial neural networks to the structure and function of the human brain underscores their utility in handling vast and complex datasets that exceed the processing capacity of humans. Another advantage is that DL models improve performance, as they are exposed to more data [20].

Given these foundational principles, the transition from general-purpose AI to domain-specific genomics frameworks becomes increasingly intuitive. The promising results of applying DL algorithms in various areas of study have led to the development of dedicated platforms using this technology in genomics, a field where researchers encounter highly complex, multi-dimensional data. There are numerous DL models used for genomic data analysis and others are emerging. Artificial neural networks resemble the biological model of the human cortex, both in terms of architectural design and function. DL models are trained using a process named backpropagation based on mathematical calculations that adjust the parameters or weights relating to prediction errors [52]. DL is also divided into supervised, semi-supervised, and unsupervised learning. There are different applications of these types of learning. For example, deep feed-forward neural networks (DFNNs) aim at solving tasks such as classification or regression performed by mapping input data to a representation and use supervised learning. Semi-supervised learning is applied when there is only a small number of data points labeled which will be used to label the unlabeled data. Unsupervised learning is commonly involved in single-cell sequencing data analysis, reducing dimensionality and clustering cells, basically identifying patterns in data without supervision [52]. Genomic studies such as cell-type classification using single-cell RNA-seq data have widely used semi-supervised learning methods with deep neural networks (DNNs) [53].

As these learning paradigms diversified, specialized architectures emerged to address specific biological problems. There are different types of DNNs used in gene regulation studies, the most popular being multi-layer perceptrons (MLPs). MLPs receive input data and direct them through a variable number of hidden layers. Other major deep-learning architectures in gene regulation research include convolutional neural networks (CNNs), recurrent neural networks (RNNs), graph neural networks (GNNs), and transformers, each presenting certain properties that prove their utility in the domain [53]. RNNs such as gated recurrent units (GRUs) or long short-term memory (LSTM) can handle long-range interactions of sequential data. RNNs present hidden states within the network which “remember sequential information at earlier locations” [15,54], being the equivalent of the memory of the network [54]. Transformers can be used for self-supervised learning on biological sequences [19]. Equipped with these architectures, researchers began applying them directly to regulatory genomics, leading to the first generation of sequence-to-expression models.

The implementation of DL models to infer gene expression patterns promises to help with deciphering existent problems in genetics research. It might also lead the way to uncovering new approaches in terms of phenotype prediction. Many of these models use DNA sequences as input rather than observed genetic variants, learning from the sequences and then being able to recognize patterns and predict outcomes for arbitrary sequences. The principle behind the learning algorithm is recognizing motifs (short repetitive patterns of nucleotides) and the elaboration of predictions is based on combinations of such motifs. This is known as the “sequence-to-expression” model. Multiple DL models were developed using this principle, the most relevant examples being Enformer [55], ExPecto [56], Xpresso [57], and Basenji [58], all of them performing gene expression prediction directly from the sequence [55].

### 3.2. Deep Learning Frameworks for Sequence-to-Expression Prediction

**Figure 1:**
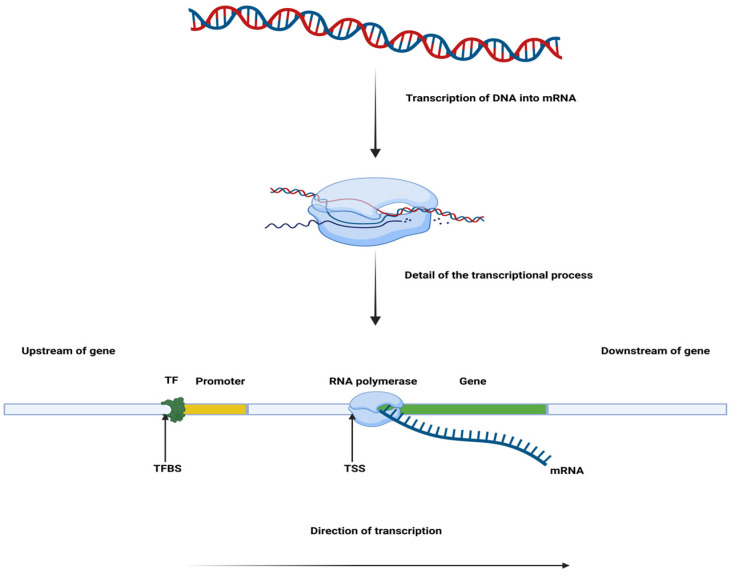
A simplified representation of transcription; the main elements that are used in gene expression prediction by the frameworks mentioned in the paper. TF, transcription factor; TFBS, transcription factor-binding site; and TSS, transcription start site. Created in BioRender. Palastea, E. https://BioRender.com/dvtafpy (accessed on 29 December 2025).

The earliest DL frameworks used for gene expression analysis operated on relatively short input sequences, under 500 bp length. They were capable of capturing local features with spatially invariant patterns among these sequences, and the architecture that best suits this kind of approach is CNN. Tools like DeepBind [59] and DeepSEA [60] predict DNA- and RNA-binding specificities of proteins (see Figure 1). They were designed to present a convolutional layer, a rectification layer, and a pooling layer. These three layers are described as a CNN block, followed by a fully connected layer. Nowadays, longer genomic regions, up to 200 kb, long enough to contain most determinants of gene expression, can be subjected to DL modeling [61].

Many later deep-learning models adopted CNN architectures (Basset [62], DanQ [63] (CNN + BiLSTM), CpGenie [64], DeFine [65], Basenji, Expecto, Xpresso, Enformer (CNN + transformer), BPNet [19], and others). DanQ, for example, has effectively employed a hybrid architecture of CNN and bidirectional LSTM (BiLSTM), outperforming DeepSEA, despite being trained on the same dataset [63]. All of the previously mentioned methods regard the genome as linear sequences per chromosome (see Table 1).

As the field continued to evolve, researchers increasingly turned their attention towards modeling 3D genome architecture and extending predictions across species with frameworks such as Akita [66] and Orca [67,68]. Orca is a tool designed for predicting 3D genome architecture from sequence-dependent features of structures such as chromatin compartments and topologically associating domains (TADs). It operates on sequence data spanning from kilobases to entire chromosomes [67].

**Table 1:** The table summarizes short descriptions of the datasets used and the output of each deep learning model mentioned.

Deep Learning Model	Target Analysis	Prediction Output	Advantages	Disadvantages
DeepSEA [56,60]	Chromatin features such as TF binding and histone marks from large-scale chromatin profiling data	DNase hypersensitivity, TF-binding sites, and histone modifications	Simple CNN architecture, directly learning from sequence (automated learning) Scalability Predictive power Good performance on large datasets	Computational costs, expensive resources High-quality demands for training data Interpretability—black box limitations available for all DL models
DeepBind [26]	Protein binding to DNA/RNA (TFs, RBPs; works on both microarray and sequencing data)	Probability for TFs/RBPs of binding on sequences prediction of sequence specificity of protein binding	Simple CNN architecture with automatic feature learning Efficiency Performs well in predicting DNA/RNA protein-binding sites Efficiency in analyzing complex patterns	Costs and resources Black box configuration The simple CNN architecture has troubles capturing long-range genomic interactions
Basenji [58]	Epigenetic and transcriptional analysis	Quantitative prediction of gene expression profiles and epigenetic marks (CAGE, TFBS, and histone modifications)	CNN architecture, but capable of analyzing longer sequences, provides information on distant enhancers Noise reduction Multitasking	Struggles with very long-range interactions, lower resolution compared to transformer-based models Analyzes the regulatory processes, but does not interpret the effect of the mutations in coding regions
Basset [62]	Chromatin accessibility, such as DNase hypersensitivity	Binary classification for chromatin accessibility in 164 cell types using sequencing data (DNase-seq)	A CNN capable of multitasking Prediction of variant effect Highly accurate predictions compared to traditional ML algorithms Automated feature extraction Pretraining accelerates the learning of new data	Designed only for processing short sequences It is more limited due to the binary prediction, characteristic of the original CNN architecture, compared to Basenji Data dependency
Enformer [55]	Epigenomic profiles and gene expression	Predicts transcriptional activity and chromatin features across large genomic regions	Transformer-based model capable of analyzing large genomic sequences Captures enhancer-promoter relationships Variant effect prediction Infers cross-gene correlation	Often fails to correctly attribute the direction of the effect of a mutation (failure to assess cross-individual correlation) High computational resources Trained on a fixed set of data, hard to generalize Limited resolution
DanQ [63]	TFBS prediction, mapping chromatin accessibility and histone marks	Predicts chromatin features and TFBS at base level	Hybrid architecture, both CNN and BiLSTM More precise than DeepSEA in predicting certain regulatory markers Predictions directly from DNA sequence Captures local and long-range dependencies	Computational expenses Needs training on large sets of labeled data to obtain accurate predictions Black box limitations
GraphReg [68]	Gene expression via 3D chromatin structure	Makes use of GATs to incorporate chromatin interaction graphs	GAT that uses 3D chromatin data to infer superior predictions on enhancer–promoter interactions Captures massive long-range interactions Higher accuracy in the prediction of TFBS	Data dependency Limited resolution Sensitive to noisy data
ExPecto [56]	Infers cell-type specific predictions for gene expression levels directly from DNA sequence based on histone marks, TF binding, and chromatin accessibility mapping	Epigenomic effect prediction using DNA sequences; predicting cell-type specific gene expression and effects of genomic variants	Directly from sequence predictions Tissue-type specific predictions Variant effect prediction Generalization across any human population variant	Limited to short DNA sequences Lower accuracy than benchmark models Fails to explain cross-individual variability Fails to attribute the right direction of variant’s effect Complex architecture
Xpresso [57]	Analyzing the sequence elements located within +/− 1500 bp around a TSS (TFBS, TSS, and chromatin accessibility) can reveal the expected mRNA abundance for the target gene	Predicts transcriptional activity (mRNA abundance) using TSS annotations and CAGE	DL model that predicts mRNA abundance directly from DNA sequence Can infer significant predictions based only on promoter sequences and mRNA stability Simple structure Efficient Can quantify non-promoter contributions to gene expression	Narrow genomic window Incorrect attributions of the direction of the variant’s effect on gene expression (increase or decrease) Limited interpretability (both due to fixed training dataset and the restricted analysis of the model)
DeFine [65]	Prediction of cell-type-specific DNA binding of TFs based on TF ChIP-seq data	Classification of TF-DNA binding or unbinding in the context of a genomic variant and prediction of the functional effect of the altered sequence	Multi-modal integration—DNA sequence, chromatin accessibility, and histone marks High accuracy of TFBS specificity prediction Captures long-range interactions	Data dependency Limited resolution High computational resources demanded Interpretability

Abbreviations: CAGE, cap analysis of gene expression; GATs, graph attention networks; RBP, RNA-binding proteins; TF, transcription factor; TFBS, transcription factor binding sites; TSS, transcription start site.

Dalla-Torre et al. introduced nucleotide transformer (NT) [69], a family of transformer-based foundation models which are pre-trained on DNA sequences from multiple genomes. It brought a different approach from the usual training of DL models, which was based on one reference genome. This initiative was conducted in an effort to overcome the poor performance of DL systems in predicting individualized gene expression patterns. The study demonstrated that NT outperforms other methods in predicting splice sites, histone modifications, and gene regulatory elements, and can even identify functionally important variants. For personalized gene expression prediction [55], paired DNA sequence and gene expression data were used as input to a specialized downstream transformer model. The reason behind training this single model by feeding it with the embeddings from a large set of genes is the theory that it might capture information that explains variation in gene expression across individuals and across genes. The performance was shown to be improved as more attention layers were added to the transformer blocks that are integrated within the model’s architecture [69].

Enformer [21], a pre-trained epigenomic model, is still considered the reference in terms of DL frameworks performing gene expression prediction. Its workflow is different from that of the NT, inferring separate predictions for each haplotype. In single-cell gene expression prediction analysis, the model captures cell-specific gene expression and enables variant effect prediction at the scale of single-cell and single-base pair. Thus, the heterogeneity of variant effect helps with the initiative of cell-type annotation and with deciphering genetic variation [21]. Enformer can capture variation across genes, but struggles with cross-individual variation, showing an average correlation of roughly zero for all tissues. Compared to traditional statistical models such as PrediXcan [55], which uses the regularized linear regression, Enformer often failed to offer a valid attribute to the variants’ direction of effect but was more exact in predicting the magnitude of the effect [52,70]. Enformer is the first DL model to embrace a hybrid architecture for gene expression and epigenetic feature prediction, having been structured on both CNN and transformer artificial neural networks [19].

Traditional linear methods still play a complementary role. Elastic net linear regression [52,71] predicts gene expression levels from SNPs (single nucleotide polymorphisms) and remains useful in transcriptome-wide association studies.

Another approach involved in the study of genomic data is the mapping of expression quantitative trait loci (eQTL). Due to the high dimensionality of genomic data, the challenges of mapping eQTL are supposed to be overcome by the emergence of ML systems like random forests [72], which is an ensemble learning method. Random forests can perform classification and mean regression of the individual trees after building multiple decision trees. Another method for performing linear regression to simplify eQTL analysis is lasso [73], which favors solutions with fewer parameter values. Predictions of DeepSEA were shown to prioritize functional non-coding regulatory mutations in Human Gene Mutation Database (HGMD) and eQTL in the GRASP search tool [19]. These improvements to eQTL mapping present the potential to infer gene expression prediction from genetic variant genotypes [72].

### 3.3. Models Focusing on Epigenetic Analysis

**Figure 2:**
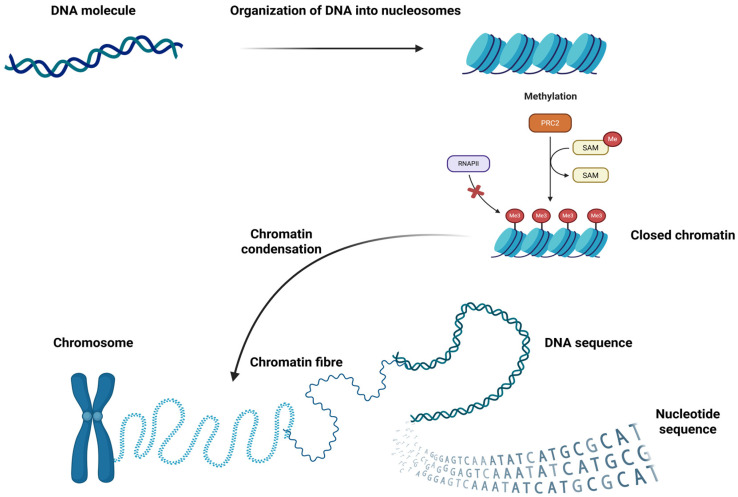
This figure depicts one of the most important aspects of chromatin organization, the closed chromatin state achieved by DNA methylation and histone methylation. This process is very important in gene expression studies. The prediction of DNA methylation status is the main focus for several deep learning frameworks described in this paper. Me, methyl group; PRC2, polycomb repressive complex 2; RNAPII, RNA polymerase II; SAM, S-adenosyl methionine. Created in BioRender. Palastea, E. https://BioRender.com/iifgvui (accessed on 29 December 2025).

Beyond variant interpretation, several models predict epigenetic modifications. CpGenie [64] predicts DNA methylation status using input DNA sequences (see Figure 2). Training for this model was performed on restricted representation bisulfite sequencing (RRBS) and whole-genome bisulfite sequencing (WGBS) profiles from ENCODE database. DeFine [65] specializes in TF-binding prediction, by training on cell-line-specific genomes instead of the human reference genome sequence. Another framework, called REUNION [74], extracts information from single-cell multi-omics data for TFBS prediction on a genome-wide scale. Multi-omics single-cell data gathering and analysis is also performed by a relatively new pipeline called MultiSC [75], developed by Lin et al., and based on a multimodal constraint autoencoder (see Table 2).

Recently conducted studies aim to integrate genomic and transcriptomic predictions, such as promoter activity and gene expression levels. Models such as Expecto and Xpresso are also gene expression level predictors. Expecto is, in fact, a redesign of DeepSEA in the form of a three-stage model, and Xpresso is projected to evaluate the mRNA expression levels based solely on the genomic sequence surrounding the promoter. Another approach is that of DeepMEL [79], constructed especially for the profiling of chromatin accessibility. BPNet [19], built as a ten-layer CNN, provides base-resolution-binding affinity predictions for genomic sequences, being trained on four TFs.

### 3.4. Models for Transcriptomic Analysis of Gene Expression

The transcriptomic-level applications are of utter importance, pinpointing the central role of the transcriptome in the gene regulation process. The diversity of the eukaryotic transcriptome emerges from the multiple levels of regulation of the transcription, thus affecting the rate of gene expression in various ways. Essential aspects that should be assessed in this kind of analysis are the existence of multiple promoters for a gene which produce RNA transcripts with different 5′-UTRs, the RNA splicing regulation process, the possibility of existence of multiple polyadenylation sites (PASs) which produce different 3′-UTRs, and others. The majority of the DL models developed for transcriptomic-level predictions use a CNN architecture. CNNProm [80], DeeReCT-PromID [81], and DeeReCT-TSS [80] are designed mainly for promoter recognition, while other applications perform splicing predictions, especially of cassette exons (DARTS [82], SpliceAI [83], and Pangolin [84]). PAS quantification can be handled by DeeReCT-PolyA [19], APARENT [19], and DeeReCT-APA. There is also the possibility of using programs that predict subcellular localization, like RNATracker, or those that predict microRNA targets—MiRTDL [85]. Additionally, DeepBind can be employed for sequence-based RNA-protein-binding prediction. Other potential research directions in the deep learning model predictions in omics are represented by the proteomic and phenotypic-level applications [19].

### 3.5. Models Specifically Used for Single-Cell Studies

Most of the aforementioned models have been trained on bulk-sequencing data, which somewhat limits their ability to capture the heterogeneity found in tissues or organs subjected to genetic analysis. Single-cell transcriptomic analysis has paved the way to understanding the differentiation during development, regeneration, and disease, but lineage tracing (which assigns single-cell transcriptomes to cellular lineage trees) remains technically demanding. Single-cell technologies also generate high-dimensional, complex amounts of data that challenge even the traditional computational approaches, making them unfeasible. This explains the tendency towards upgrading from traditional statistical models and ML to DL alternatives [52].

ML algorithms have been introduced for identifying cell lineages from scRNA-seq data. A computational tool already used in this attempt is GEMLI [86] (gene expression memory-based lineage inference). GEMLI facilitates the study of heritable gene expression and is able to perform discrimination of symmetric and asymmetric cell fate decisions and reconstruction of multicellular structures from pooled scRNA-seq datasets. GEMLI can be applied to any scRNA-seq dataset, only requiring exonic reads, and is based on the characteristic expression distributions of memory genes across cell types. It is best suitable for analyzing only small to medium-sized cell lineages related over several generations, but will perform less robustly in cell populations that contain distantly related cells [86]. Another algorithm, available as an R package (compatible with R version 4.1.3 or higher), for predicting TF-gene interaction using scRNA-seq is scGATE [87].

One application was to infer single-cell gene expression profiles from TF analysis using a tree-guided multi-tasking approach, a framework used by multiple models such as TRIANGULATE [88] or SCENIC [88]. They have a similar concept, deriving a TF activity score per cell and studying the associations between single-cell gene expression and TFs. These statistical models are trained to consider the gene expression measurements of genes across single cells as being the tasks in the MTL set-up.

Further work in this field was directed towards integrating DL resources. Efforts have been undertaken in an attempt to fill in the missing temporal information of single-cell gene expression. Current techniques do not enable researchers to monitor the profile of scRNA-seq data continuously over time, therefore performing measurements at discrete time points. A realistic in silico prediction at any time is needed in order to have a more exact representation of the dynamics of gene expression. One framework which attempts to predict accurate gene expression at any time point is scNODE [89], which combines a variational autoencoder (VAE) with neural ordinary differential equation (ODE) [89]. A desired approach is using statistical methods for approximating ODEs that serve for modeling gene regulatory networks (GRNs) [90].

Several computational approaches based on DL algorithms have emerged lately for the various stages of scRNA-seq analysis. Most of them focus on normalization, data correction, and downstream analysis steps. Removing technical artifacts or unintended effects requires detecting and removing changes in measurements between samples and features through normalization. DL models designed for this task include DCA [91], SAUCIE [92], Auto Impute [93], DeepMc [94], Deep Impute [95], and scVI [96]. Further research on this topic intends to overcome the challenges of technical noise from scRNA-seq data.

Data correction algorithms such as ResNets [97], MNNs [97], BERMUDA [98], DESC [99], and others promise to account for variables represented by batch, dropout, and cell cycle effects. Dropout occurs when a gene is observed at a moderate or high expression level in one cell but is not observed in another cell [52,100]. Imputation methods for scRNA-seq are divided into two categories. The first type includes those who change all gene expression levels (e.g., MAGIC [101]) and single-cell analysis via expression recovery (e.g., SAVER [102]), while the second category is based on methods that impute dropout events, such as scImpute. Most algorithms developed for dropout events identification are based on AEs (autoencoders) and the examples are AutoImpute, Deep Count AE Network (DCA), and SAUCIE. Other levels where DL algorithms can analyze scRNA-seq data are dimensionality reduction (scvis [103], BasisVAE [104], and SAUCIE), clustering, and cell annotation (DESC, scDCCA [105], and scDeepCluster [106]). Cell–cell communication can be inferred using programs such as CellChat [107] or CellPhoneDB [108]. Multi-modal integration is possible with models like scMVAE [109], DCCA, and DeepMaps [110]. DeepVelo [111] is a platform that uses RNA velocity, a technique evaluating a cell’s fate based on the ratio between newly synthesized, unspliced mRNA and mature mRNA, for dimensionality reduction in the dataset [111].

Gene expression is subject to certain changes in the context of external influences. Single-cell gene expression studies can be used to explore cellular responses to infection, gene editing, and drug exposure. Understanding these effects at single-cell resolution may provide deeper insight into future clinical, medical, and pharmacological approaches. The difficulties of obtaining perturbed tissue samples and inferring a prediction from this analysis require the use of bioinformatics tools, of which DL models have a promising outlook. One of the most recent initiatives in this area uses scPRAM [112], a model for predicting perturbation responses in single-cell gene expression based on attention mechanisms. scPRAM can infer an accurate prediction of gene expression responses to perturbation for unseen cell types after aligning cell states before and after perturbation. The VAE used for this model reduces the high dimensionality and data sparsity of single-cell gene expression data, mapping this matrix to a lower-dimensional latent space [112]. Other similar approaches and deep learning models designed for the same applications are scVIDR [113] and scGen [114], also using VAE, but in different manners. Nonetheless, they appear to have a lower performance than scPRAM. Another model employed in the study of perturbation’s role in gene expression prediction in single-cell techniques is CellOT [115], having a different architecture from the already mentioned frameworks. It integrates optimal transport and input convex neural architectures and seems to be very efficient in predicting single-cell drug responses. CellOT directly learns and uncovers maps between control and perturbed cell states and then predicts perturbation responses [115].

Attention-based neural networks are introduced in the development of deep learning predictive models applied for phenotype prediction. One model with this architecture is ScRAT [116] which uses scRNA-seq data for training. ScRAT consists of the following three modules: sample mixup, attention layer, and phenotype classifier. It can learn from a limited number of training samples and is independent of cell-type annotations, being a promising tool for finding phenotypic-driver cell types that can lead to the discovery of novel molecular mechanisms and targeted therapies.

### 3.6. The Potential of Quantum Computing Approaches for Gene Expression Prediction

Quantum computing and its intersection with ML are emerging computational paradigms that are being investigated for their applicability to high-dimensional biological data analysis. Although most applications remain in early, proof-of-concept stages and are not yet clinically deployable, these methods have the potential to become complementary tools for classical bioinformatics techniques [117].

One representative example is the development of a quantum circuit model for inferring GRNs from single-cell transcriptomic data derived from human lymphoblastoid cells [118]. Quantum models use a basic unit of information to encode data, named qubit. In this quantum single-cell GRN (qscGRN) model, each gene corresponds to a qubit. Leveraging phenomena like superposition and entanglement, scRNA sequencing data are used to determine regulatory gene–gene dependencies within a quantum framework [118]. In addition to circuit-based models, quantum annealing approaches have been explored for optimization tasks, including single-cell RNA-seq data clustering. Alternative low-energy solutions could be obtained and may facilitate exploration of different gene grouping configurations [119].

Beyond clustering, other studies have explored the comparative capabilities of quantum machine learning (QML) versus classical frameworks for gene expression classification, assessing pattern extraction, feature relevance, and computational constraints [120]. Furthermore, quantum machine learning models, through quantum kernel methods, have been evaluated for breast cancer molecular subtype classification, showing performance comparable to classical approaches while requiring fewer data points [121].

Complementing these classification-focused efforts, other quantum frameworks focused on the identification of attractors in GRNs modeled using Boolean networks. Because the number of possible network states grows exponentially with system size, identifying these attractors (stable states associated with biological phenotypes) is computationally challenging for classical methods. To address this limitation, quantum search strategies have been proposed for attractor identification in synchronous Boolean networks, indicating resilience to noise on current noisy intermediate-scale quantum (NISQ) devices [122]. Altogether, although these approaches remain in the proof-of-concept stage, they provide early evidence that quantum techniques could offer complementary perspectives for the analysis of complex gene expression data.

## 4. Insight into the Role of Spatial Transcriptomics in Phenotype Prediction

There are multiple neural networks architectures that are currently used for genomic studies. Each of them is developed according to certain characteristics and designed for specific purposes. For example, MLPs, RNNs, and CNNs are frequently used in these research areas. RNNs perform sequential data analysis for text, speech, or DNA sequences, while CNNs can draw spatial relationships from image data. CNNs use convolution filters to extract granular features from a traversed image. Such applications suggest the potential of these neural networks to aid in histopathology and genomic diagnostics. Certain CNNs have already been proven efficient in predicting a few types of cancer based on digital histopathology [123].

Building on these methodological strengths, incorporating spatially resolved molecular data has become essential for placing gene expression back into its native tissue context. Spatial multi-omics technologies might be key to the characterization of a sample, which is usually a fixed fresh-frozen or formalin-fixed and paraffin-embedded tissue section that can also be stained. Most of the time, hematoxylin and eosin (HE) staining is used. When obtaining sections from the target tissue, serial sections can be specifically dissociated into single cells or nuclei which will be subjected to single-cell sequencing. Data obtained from this analysis will serve for the optimal deconvolution of spatial information and additional data integration. Apart from the previously listed methods of performing single-cell sequencing and analyzing the results, including the DL models involved in the analysis of the sparse data, there are also integrative methods which additionally perform the association with tissue morphology. Some rely on microscopy-based methods, such as single-molecule FISH (smFISH) (multiplexed error-robust FISH (MERFISH [124]), sequential FISH (seqFISH [125]), and OligoFISSEQ [126]), while others use laser capture microdissection-based isolation of single cells from the sections and techniques of single-cell sequencing (epigenomic, transcriptomic, proteomic, etc). MERFISH is based on a system where each gene has an associated binary code, where 1 signifies fluorescence and 0 means no fluorescence. seqFISH relies on a system that assigns a color sequence code for each gene, having twenty-four color probes per gene and sixty different pseudo-color options [127]. The integration of data generally requires different computational models which can deal with the large amount of sparse genomics data, and the current focus is on DL models that use various types of neural networks (VAE, adversarial autoencoders—AAEs, ViT, and LSTM) [128].

ST offers the opportunity of studying gene expression patterns while preserving the spatial configuration of the intact tissue [9]. Applications developed for ST mapping of cell types are Bayesian models, such as Cell2location [129], which uses scRNA-seq reference of cell types integrated with 10x Visium, having a 55 μm resolution and roughly 3–30 cells per capture spot to spatially resolve fine-grained cell types. ST techniques such as Visium and Slide-seq [130] present the disadvantage of their resolution being far lower than single-cell level, each pixel in Slide-seq covering multiple cells (~10 microns), while Visium spots average around 10–20 cells for each 50 microns. Consequently, the measured gene expression at a spot reflects a mixture of cells [131].

The main focus of research regarding gene expression prediction is the design of methods of studying and obtaining omics data without damaging the tissue, preserving its initial architecture and pattern of gene expression. Another important target would be inferring such data from viable, living cells, providing information from a dynamic point of view. These methods attempt to extract features that are much closer to the real-time events happening within a tissue or organ.

A useful modality to monitor live cells is offered by Raman microscopy, “a unique tool that relies on the collection of vibrational energy levels of molecules” [13]. Raman microscopy has the advantages of being nondestructive and label-free but lacks genetic and molecular interpretability. Recent research by Kobayashi-Kirschvink et al. introduced a computational framework named Raman2RNA (R2R) [13] that can infer single-cell RNA expression profiles from label-free nondestructive Raman hyperspectral images. The approach integrates single-molecule fluorescence in situ hybridization (smFISH)-anchor-based integration data of selected markers from the same cells, and scRNA-seq profiles to train the model. Prediction of scRNA profiles can also be performed in an anchor-free manner with AAEs [132]. The final step is the translation of Raman images into single-cell expression profiles. R2R ultimately offers a label-free live-cell inference of single-cell expression profiles over time, thanks to tracking live single cells by time-lapse Raman imaging. Briefly, R2R could provide expression profiles at single-cell resolution by using multiplex smFISH as an anchor between the images and the scRNA-seq profiles [13].

ST plays an increasingly important role in today’s research in gene expression prediction, as it mixes gene expression data with the localization of cells in a tissue. It unveils aspects regarding cell communication and interaction in their natural environment. On a single-cell scale, it further develops an improved image of the tissue heterogeneity and the cooperation and function of each cell inside a particular organ or tissue. Besides Raman, plenty of ST-focused frameworks have been implemented. The use of DL applications in this field is of utter interest, considering the complexity and quantity of the extracted data, which sometimes exceeds the human analysis capacity.

To overcome these analytical limitations, several neural frameworks now explicitly integrate patch-level morphology with spatial gene expression matrices. TISSUE [132] is a DL framework implemented as a spatial gene prediction model, relying on ST and scRNA-seq data. It estimates uncertainty for spatial gene expression prediction for unmeasured transcripts and is based on supervised learning. Histology images used as a support for inferring gene expression patterns present a growing interest, considering the reduced costs of slide preparation compared to the complexity of other protocols for similar purposes. HGGEP [133] is a hypergraph neural network model developed for predicting gene expression levels from histology images. HGGEP partitions the slide in multiple patches centered around spots. Cell morphological information perception of the model is enhanced by integrating a gradient enhancement module (GEM). The modular structure employing attention mechanisms and LSTM enables this model to refine spatial information and produce detailed gene expression landscapes [133]. To further exploit the link between tissue morphology and gene expression, Pham et al. created stLearn (version v1.2.2) [134]. This software was designed for identifying cell types, observing cell–cell interactions and the influence neighboring cells inflict upon transcriptional processes at their location in the tissue. This framework integrates a pseudo-time-space algorithm that traces the changes in gene expression over time across tissue regions, which can reveal links between tumor progression and local responses [134].

Prediction of gene expression from whole-slide images (WSIs) data is gaining more and more interest from the research field, introducing methods such as ST-Net [135], a DL model that captures high-resolution gene expression heterogeneity from histology slides and ST data. Pizurica et al. worked on a DL framework capable of leveraging gene expression predictions from histology slides. Their application, named SEQUOIA [136], focuses on prediction of cancer-associated genes and uses a DL architecture with linearized attention, performing a digitalized histopathological interpretation. Other DL programs designed for similar purposes are HisToGene [137], DeepPT [138], Hist2ST [139], and THItoGene [140]. WSI morphological features have been associated with genomic mutations [141], gene expression profiles, and methylation patterns [142].

Deep generative models have also emerged. They are used to generate WSI tiles after being infused with matched gene expression profiles. Carrillo-Perez et al. designed a model called RNA-GAN that utilizes a VAE to learn a latent representation of the multi-tissue gene expression profiles which is then transferred to a generative adversarial network (GAN). GAN generates synthetic tissue tiles based on the learnt information [143]. Expanding beyond generative approaches, Zheng et al. identified a particular role of artificial intelligence in predicting immune and inflammatory gene expression signatures from hepatocellular carcinoma histology, proving a possible diagnostic purpose [144]. Building on this momentum, Zhao et al. developed SpiRiT, a vision transformer (ViT) framework to infer spatial single-cell transcriptomic signatures from HE-stained histology slides and named it SpiRiT [145] (Spatial Omics Prediction and Reproducibility Integrated Transformer). This model was tested for predictions for human breast cancer and whole mouse pup histology slides [145].

Some initiatives to increase the resolution of ST to single-cell level have emerged and they rely on AI computational models. SPOTlight [146] is a deconvolution algorithm built upon a non-negative matrix factorization regression algorithm and least squares regression to determine a spot’s composition, defining cell-type-specific topic profiles and the characteristic gene distribution of a cell type. It enables an automated interpretation through the possibility to integrate unpaired ST and scRNA-seq data. The limitations consist of the ineffective learning and use of the intrinsic topological information of cell-type constitutions within spots. A potential tool to overcome these challenges seems to be the development of semi-supervised models based on graph convolutional networks (GCNs), in which intrinsic topological information can be represented as graphs. Deconvoluting ST data through graph-based convolutional networks (DSTGs) models have shown good results, are based on scRNA-seq datasets, and are able to learn the precise composition of ST data using a semi-supervised GCN [131].

Another model, STEM [147] (SpaTially aware EMbedding), applies deep transfer learning to jointly encode both ST and single-cell data into a shared space, allowing pseudo-spatial mapping between cells. This approach compensates for the lack of spatial resolution in single-cell methods and for the limited coverage of ST techniques.

scResolve [148], developed by Chen et al., is a method for recovering single-cell expression profiles from ST measurements at multi-cellular resolution, restoring expression profiles of single cells at their specific location, a task which cannot be performed by deconvolution. scResolve combines spot-level expression profiles with the paired histology slide image, resulting in subcellular gene maps. It then segments these maps into individual cells and produces expression profiles. Segmentation is performed firstly by identifying the nuclei from tissue-staining images; then, a DL transformer model infers, for each spot from the gene expression map, if it is a part of a cell or of the extracellular matrix and its relative position related to the center of its nucleus. Finally, the spots identified as parts of the cells are grouped according to the relative positions to nucleus centers.

Stellaris web server [149] offers fast and accurate spatial mapping of user-uploaded scRNA-seq data. Many additional AI applications, such as SOMDE [150], scGCO [151], SEDR [152], coSTA [153], STAGATE [154], and GCNG [155], support ST analysis but do not reach true single-cell resolution. Single-cell resolution was achieved with ST data by using NCEM [156].

ST has shed light on multiple aspects of omics, solving problems related to the dimensionality of data and providing a holistic approach to gene expression. Another factor that contributes to reducing the bias of transcriptomic gene expression characterization in ST data is retaining the spatial configuration, as it does not need tissue dissociation. This helps with providing the tissue context that is very relevant for understanding the signaling between cells at a known location [9,134]. The advancement towards single-cell resolution certainly increased the performance of the techniques by deconvolution and referencing single-cell datasets. Morphological identification relying directly on pixel-level analysis might further reduce the error occurrence observed in the single-cell segmentation. Limitations still occur, with many AI models employed for such tasks failing to identify consensus tissue domains (TDs). In an attempt to improve clustering of cell types based on similarities in gene expression patterns or molecular functions, Kaur et al. proposed MILWRM [157], an algorithm designed for TDs characterization using multiplex immunofluorescence and ST data.

## 5. Translational Implications

AI applications have been introduced in the clinical settings, gradually becoming a powerful tool for healthcare providers. No-code AI platforms, such as the one described by Hoseini et al. [158], are very useful for people that do not have expertise in coding and can perform tasks such as white blood cells classification. Other AI frameworks are tested for dental medicine applications [159], boosting diagnostic accuracy, treatment, and monitoring through teledentistry [160]. The great enhancement in interpreting imaging data brough by AI also encouraged initiatives to implement such models in cardiovascular imaging [161]. Further research for fine-tuning these models targets optimization of microscopy techniques and nanoscale imaging, as it is described in the work of Zhao et al., where they used ML for expansion microscopy samples from clinical specimens [162].

As these clinical applications multiply, single-cell and spatial omics technologies are beginning to reshape the landscape of translational research by providing unprecedented molecular detail. Single-cell omics technologies uncover tissue and organ heterogeneity at an unprecedented scale and have shown promising results in conjunction with various AI programs for predicting gene expression. Clinical application should certainly benefit from the thorough analysis that can be performed by these innovative methods. The huge ability of single-cell techniques to dissect tissue heterogeneity has impacted the way of analyzing malignancy and certainly helped with understanding the processes that govern the tumoral microenvironment and cell communication at unprecedented resolution. The details revealed by using these techniques have brought new insights in studying targets, drugs, and molecular pathways that play important roles in producing pathologies [163]. Oncology is one of the fields that will massively use the evolution of single-cell sequencing and AI algorithms for diagnostic and treatment purposes, taking personalized medicine a step further. There have already been initiatives that integrated such technologies, with Fan et al. using ML algorithms for analyzing single-cell RNA-seq data for osteosarcoma, particularly targeting CD8^+^ T cells [164].

Zhao et al. applied their work with SpiRiT [145] to improve targeted therapy strategies, integrating ST data with the histopathological landscape of tumors, to provide a more precise map of gene expression. SPiRiT predicts spatial gene expression profiles at a single-cell resolution for human breast cancer and also works for multiple species and tissues. Patient outcome in oncology can see important improvement in the future after implementing these tools in cancer therapy and diagnosis [145].

AI modeling promises not only to enhance the drug development and diagnostic area, but it also aims to predict the outcomes of different diseases. Personalized medicine holds the premises for uncovering the underlying mechanisms of diseases in individuals in a patient-tailored manner. AI- driven models may soon ensure dynamic risk prediction, allowing the anticipation of disease progression or treatment response based on leveraging genomics and transcriptomic signatures. The role of minor mutations may be established thanks to these frameworks, such as the influence of SNPs on drug susceptibility in different patients. One of the main goals of AI-based omics analysis remains the identification of clinically relevant variants. In clinical practice, promising models are being adapted for patient monitoring, selection of candidates for clinical trials, and risk stratification [20]. AI algorithms and models have demonstrated powerful clustering capacity, proving to be increasingly useful for patient risk stratification based on selected biomarkers. For example, applications for sepsis phenotyping speed up the diagnosis, predictions for clinical outcome of the patient, and bring new insights into optimization of treatment thanks to the analysis of drug response, dosage in different sub-phenotypes, and potential drug targets [165,166,167]. Such frameworks can integrate clinical parameters and omics data for a thorough selection of relevant information used in the predictions.

In parallel, spatial and single-cell deconvolution methods are being tested directly on patient tissues to identify cellular niches and subpopulations linked to clinical behavior. DSTG [131] was tested on pancreatic ductal adenocarcinoma sections, providing insight into its capacity of spatially mapping the prevalence of different cell subpopulations. The experiment additionally revealed the ability of detecting unique cytoarchitectures and cancer-linked phenomena.

The majority of DL systems tested for single-cell gene expression in the context of ST analysis have been applied to cerebral cortex regions and rendered a topological structure similar to the real distribution. Building on these early demonstrations of spatial fidelity, the most important applications, as mentioned above, are the oncology-related ones. Hao et al. used their tool called STEM [147] for characterization of tumor microenvironments on a single-cell scale based on a dataset of human squamous cell carcinoma. The results were comparable to manual gene selection but managed to reduce the analysis time and the error rates encountered in the human-curated study. STEM also showed promising results in retrieving gene expression patterns from liver sections and endothelial cells, analyzing spatial variation. These advances underscore how AI is becoming indispensable for interpreting complex transcriptional programs in disease.

It is worth noting that machine and DL platforms proved to be useful in the context of cancer immunotherapy. This is especially critical because cancer immunotherapy has emerged as one of the most transformative treatment paradigms in modern oncology, yet its efficacy is profoundly shaped by the cellular heterogeneity and immune landscape that only single-cell technologies can resolve. In this research area, the shift from bulk to single-cell technology has contributed to the scale at which tissue heterogeneity was observed and brought more accuracy to the predictions. The quantity of encountered data also turned the researchers’ attention to AI alternatives that can work out a solution for the rich transcriptomic information. AI approach is both able to remove technical noise and to provide novel insights into omics profiles. These algorithms can decipher the underlying mechanisms for tumor resistance to immunotherapy and also uncover the weak spots that can be attacked with treatment alternatives [168]. By illuminating these mechanisms of therapeutic resistance, AI-driven single-cell analysis provides a foundation for more rational and personalized immunotherapy strategies.

In parallel with these immunotherapy-focused advances, researchers have increasingly used DL to explore additional regulatory layers that influence tumor behavior, particularly alternative splicing programs involved in development and metastasis. Zhang et al. developed a DL framework that adds data from empirical RNA sequencing to produce predictions of alternative splicing sites. This platform is called DARTS [82] and was used for studying processes encountered in the development of embryos and cancer metastasis. In this case, because the process studied concerns the epithelial–mesenchymal transformation, the system performed best when trained on combined information from two databases, ENCODE and Roadmap [82].

**Figure 3:**
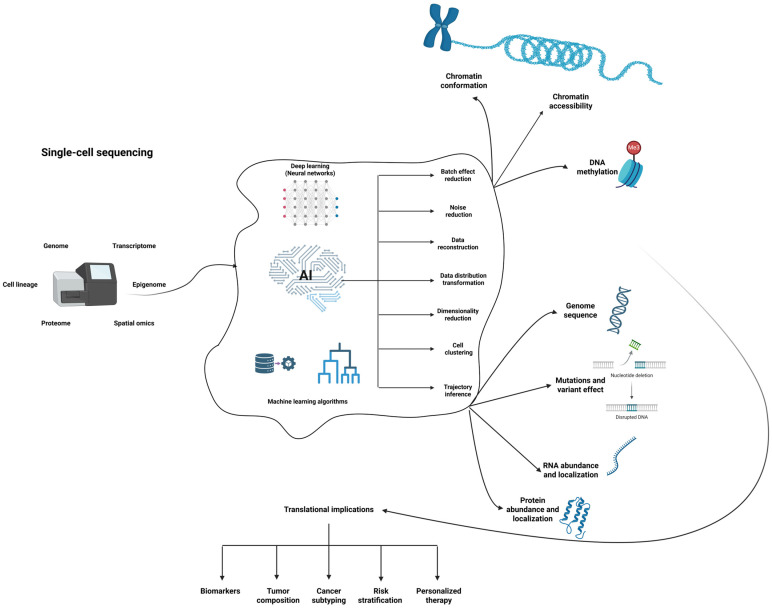
Summarizing diagram that presents a simplified workflow from single-cell sequencing to gene expression prediction using AI methods and the translational applications resulting from the inferred cell phenotypes. Abbreviations: AI, artificial intelligence. Created in BioRender. Palastea, E. https://BioRender.com/asl891t (accessed on 7 January 2026).

Other DL algorithms were designed for gene expression-based cancer prediction, such as WT-GAN [169], which operates on microarray gene expression cancer data as input (see Table 3 for a summary of the models that have translational applications). Together, these translational frameworks highlight the accelerating convergence of single-cell biology, spatial omics, and DL in modern precision medicine (Figure 3).

## 6. Benefits and Limitations

The rapidly growing interest in single-cell technologies and in developing novel computational approaches to handle the immense quantity of sequencing and spatial data are driving an accelerated evolution in these domains. The need for continuous improvement is demonstrated by the huge potential of these methods to fill the gaps in omics research and omics-related clinical approach. However, every step forward comes with inherent limitations, and the technological surge requires careful validation to define each application’s role and prevent errors (see Table 4).

For example, single-cell RNA sequencing (scRNA-seq) encountered issues regarding the temperature conditions used in the protocol. The dissociation process at 37 °C induced artificial changes in the expression profiles following the activation of stress genes transcription distorting gene expression profiles. This problem was mitigated by lowering the temperature at 4 °C during dissociation, but there may be other factors that can trigger the expression of stress genes [1]. scRNA-seq, even with the help of DL platforms, cannot offer enough details related to the spatial context from which the cells were extracted, due to the tissue dissociation process. Nonetheless, even with improved protocols, the loss of spatial context during dissociation remains a major gap in single-cell data. This limitation naturally shifts attention toward spatial methods and supports their integration with single-cell approaches for a more complete biological picture.

The single-cell technologies raise issues connected to the statistical errors that may interfere with the final results’ reading. Transcriptomic technologies have broadened the knowledge regarding the complexity of certain tissues and the social network that establishes between cells. It complements single-cell sequencing, because ST itself lacks the desired resolution. Although single-cell omics fail to preserve spatial integrity, thereby losing details, the information retrieved from spatially resolved transcriptomics combined with the single-cell techniques can produce an enhanced picture of the organization within tissues and also offer a dynamic perspective [163].

Another limitation of scRNA-seq is the clustering resolution. DL frameworks such as stLearn improve clustering by integrating spatial information and tissue morphology for gene expression prediction, using SMEclust, to find subclusters of cells that were not identified by classical scRNA-seq methods [134].

Single-cell multi-omics have paved the way to deciphering cell population heterogeneity within tissues but encountered several limitations over time, like high costs and technical difficulty. The downsides of the combined approach of single-cell multi-omics and histology slide imaging reside in the heterogeneity of the sample (not all of the omics techniques are compatible with all sample types) and the encounter of single-cell statistical uncertainty. Using histology slides for inferring gene expression prediction is a cost-reducing alternative, but nonetheless a challenging task, having its limitations in ignoring the complex relationship between cell morphology and gene expression patterns and not utilizing the full features extracted from the images. There has certainly been huge improvement, also with the aid of advanced computational methods and new technology that have emerged [171]. Nonetheless, one aspect that still restricts the number of applications for single-cell omics is the statistical part that generates computational errors. Technical limitations raise concerns when analyzing results from scRNA sequencing, as there is a high dropout rate. A dropout event is, in fact, a false “zero” result, which translates into the inability to detect an expressed gene because of a low RNA capture rate. A true zero result represents a gene that is not expressed in a particular cell type. Shorter genes present a greater dropout rate and have lower counts [52]. The need for discerning between real and false zero values has led to the development of statistical models and DL methods, but this area still deals with a lot of cases where a suitable model has not been introduced yet. Data sparsity in scRNA-seq is a challenge that can impede downstream analyses and confronts scientists with multiple statistical problems. Some of them are related to the uncertainty of whether the observed zero values from the analysis are a reflection of the absence of gene expression in the cell or they occur due to a lack of data regarding that gene [171].

Variations in scRNA-seq data stem from multiple factors, including sequencing depth, variation in cell type, and technical artifacts. To overcome such limitations, the statistical models should account for error distribution and parameter uncertainty. Models suitable for this kind of analysis often rely on Poisson distribution, negative binomial distribution, generalized linear models (GLMs), and Pearson residuals for dimensionality reduction. Whilst this approach solves statistical issues that hinder the full recovery of data from scRNA-seq, it cannot be directly applied to new single-cell platforms [172].

**Table 4:** Benefits and limitations of the discussed methods.

Methods Used	Benefits	Limitations
scRNA-seq	High resolution Reveals tissue heterogeneity Captures rare cell populations Lineage and trajectory analysis Enables understanding of microenvironment interactions	Artificial transcriptional stress responses Lack of spatial context Statistical errors Low-resolution clustering High dropout rate and false zero values Data sparsity Variations in cell sequencing depth
ST	Preserves spatial integrity Maps tissue architecture Infers spatial gene expression patterns Provides a link between transcriptomics and morphology of a tissue Suitable for pathology studies (tumors, fibrosis, immune cells infiltration, etc.)	Lacks desired resolution Low transcript detection Complexity High cost Complex interpretation tools—pipelines—for data analysis
DL platforms [173]	Can find subclusters and patterns that are not identified by classical scRNA-seq methods Efficient use of sparse, high-dimensional data Can extract non-linear relationships Can be fitted for large datasets Can integrate multiomics data Improved denoising	“Black box” configuration—hidden states of the network that often impede a trustworthy biological prediction. The complex layered architecture masks interactions between millions of parameters within the networks, rendering their decision-making process hardly comprehensible to most human observers Require a suitable dataset Require computational resources Must be fed with large labeled datasets Selection of a suitable model for the task Ethical concerns regarding sensitive data usage for training the models (data privacy) Lack of suitably structured data of the clinical information systems for feeding AI models Biased predictions produced by training on clinical data retrieved from limited cohorts of study

Abbreviations: AI, artificial intelligence; DL, deep learning; scRNA-seq, single-cell RNA sequencing; and ST, spatial transcriptomics.

Interpretation of (ST) data also raised a lot of challenges and some issues still persist, with methods like cellular segmentation and annotation being prone to error. While these techniques are gaining popularity, especially because they have been complemented by AI-powered curation lately, there are multiple aspects that are not adequately tackled by the current technologies. Many of them are unable to identify consensus tissue domains (TDs), which refer to grouping tissues or cell types based on similar gene expression patterns, molecular functions, or biological roles [157].

Particularities of both ML algorithms and DL models as AI subsets indicate the advantages and limitations of the methods. On one side, ML depends on careful data preprocessing that usually requires multiple steps to select suitable data. On the other side, DL has the advantage of automated learning directly from raw or minimally processed data. This feature allows DL to deal better with complex and non-linear data and makes the process less time-consuming. Regarding the training data, ML can usually be trained on small and medium datasets, while DL requires large datasets. DL presents the risk of overfitting, thus needing the large sets of data to train on. Thanks to the statistical and mathematical structure of ML models, these present more interpretability and are easier to follow by a human observer, while the DL frameworks have the “black box” configuration that usually prevents the observers from understanding how predictions are made by the model. This issue is one of the main reasons for reticent implementation of DL in clinical applications. A further difference can be seen in the costs, as the DL models require high computational resources and longer training times. The comparative performance of ML and DL depends on the task; DL can outperform classical ML in the case of large datasets or where complex genomic interactions are analyzed, but it does not consistently perform better. ML tends to have a more stable performance across datasets thanks to the underlying algorithms, and this feature makes it suitable for classification tasks and disease phenotype prediction [174].

## 7. Conclusions

The continued development of innovative approaches for omics data analysis has substantially advanced our understanding of cellular heterogeneity, gene regulation, and disease-associated molecular mechanisms. Omics medicine lies at the intersection of genomics and bioinformatics, showing that computational methods are essential for understanding complex biological processes. AI-based algorithms have already made a significant contribution to gene expression prediction, enabling the identification of regulatory patterns that were previously difficult to capture using traditional analytical frameworks.

Although gene regulation remains incompletely understood, ongoing refinements in ML and DL architectures, together with improved optimization strategies, continue to reduce prediction error rates. Single-cell sequencing technologies have improved data consistency and reproducibility, strengthening the reliability of computational modeling. The inclusion of spatial omics technologies, designed to extract gene expression profiles at single-cell resolution, combined with the increasingly proficient AI platforms, represent major steps toward precision medicine. Together, these integrative approaches allow us to reconstruct cellular states in multiple dimensions, revealing how transcriptional programs interact with the tissue environments that shape them.

These advances build a strong foundation for patient-tailored predictions of disease progression and therapeutic response. Continued progress in experimental technologies, complemented by emerging computational paradigms, including quantum computing, is expected to further accelerate biomedical research and deepen our understanding of GRNs underlying human health and disease.

## Figures and Tables

**Table 2:** Deep learning frameworks that predict DNA methylation at CpG sites—a short description of the main target and the prediction output for each of them.

Model	Target Analysis	Prediction Output	Advantages	Disadvantages
CpGenie [64]	CpG methylation status	Infers predictions regarding CpG sites based on regulatory regions (enhancers, promoters, etc.)	CNN architecture capable of single-nucleotide resolutions (high sensitivity) Learns directly from DNA sequence and can produce predictions for the effect of previously unknown variants Variant prioritization	Not suitable for analyzing long-distance genomic interactions Lower performance in CpG-poor regions Data dependency It is an older model and has a less accurate performance compared to modern, transformer-based frameworks
MethylNet [76]	Methylation status at CpG sites and non-CpG sites	Enhanced prediction accuracy of methylation sites using additional chromatin data	Structured on VAEs, it can perform both supervised and unsupervised learning Multitasking model Can capture nonlinear relationships between CpG islands High-fidelity data	Requires advanced computational resources Limited interpretability Sensitive to batch effects
DeepMethyl [77]	DNA methylation status (CpG and non-CpG sites)	A hybrid model (CNN-RNN) that evaluates the methylation levels across both CpG and non-CpG regions	Integrates 3D genomic data Has integrated denoising autoencoders It performs better than benchmark ML models	In the absence of data about methylation status of the neighboring regions, its accuracy decreases Limited genomic coverage Data dependency Interpretability issues
DeepSignal [78]	CpG methylation	Distinguishes between methylated and non-methylated CpG based on nanopore sequencing data	Hybrid CNN and BiLSTM architecture which ensures a dual-feature extraction with a higher accuracy than traditional ML algorithms Very efficient for low-depth sequencing data Performs better than bisulfite sequencing	High computational demand It is outperformed by newer models Requires retraining Black box configuration limitations Data dependency

Abbreviations: BiLSTM, bidirectional long short-term memory; CNN, convolutional neural network; ML, machine learning; RNN, recurrent neural network; VAE, variational autoencoder.

**Table 3:** Translational applications for ML and DL frameworks trained on single-cell sequencing data. This table offers a short description of the architecture of the deep learning model and the potential applications for diagnostic purposes.

Framework	Architecture	Application	Reference
LASSO, XGboost, GBM, Boruta, CoxBoost, survival-SVM	ML algorithms	Predicting T cell exhaustion signature from genes in osteosarcoma	Fan et al. [164]
SpiRiT	ViT	Predicting spatial gene expression profiles for human breast cancer	Zhao et al. [145]
DSTG	Graph-based convolutional network	Deconvolution of pancreatic cancer tissue sections	Song and Su [131]
STEM	MLP encoder Transfer learning	Characterization of tumor microenvironment on single-cell scale based on datasets of squamous cell carcinoma; retrieving gene expression patterns from liver sections	Hao et al. [147]
iMAP	GANs and deep autoencoders	Removes batch effects and identifies batch-specific cells	Gui et al. [168]
DARTS	DNN and a Bayesian hypothesis testing statistical model	Useful for studying the development of embryos and cancer metastasis	Zhang et al. [82]
WT-GAN	GAN	Predicting cancer types based on MGCED datasets	Ravindran and Gunavathi [169]
Seurat [82]	ML algorithm	Characterization of epithelial cell lineages in lung adenocarcinoma	Zhang et al. [82]
PMG training framework	5 PMGs ViT-L model, Segment Anything Model, and ResNeXt framework	Differentiation between benign and malignant cells and leukemia typing	Yan et al. [170]

Abbreviations: DNN, deep neural network; GAN, generative adversarial network; ML, machine learning; MLP, multi-layer perceptron; PMG, multistage progressive multigranularity; and ViT, vision transformer.

## Data Availability

No new data were created or analyzed in this study. Data sharing is not applicable to this article.

## Abbreviations

AI, artificial intelligence; BiLSTM, bidirectional long short-term memory; CAGE, cap analysis of gene expression; cDNA, complementary DNA; CNNs, convolutional neural networks; DL, deep learning; DNA, deoxyribonucleic acid; DNNs, deep neural networks; eQTL, expression quantitative trait locus; FISH, fluorescence in situ hybridization; GANs, generative adversarial networks; GAT, graph attention network; GEM, gradient enhancement module; GNNs, graph neural networks; GRNs, gene regulatory networks; GRUs, gated recurrent units; GWAS, genome-wide association studies; HE, hematoxylin and eosin; LSTM, long short-term memory; ML, machine learning; MLPs, multi-layer perceptrons; mRNA, messenger ribonucleic acid; QC, quality control; RNA, ribonucleic acid; RNNs, recurrent neural networks; sc, single-cell; scRNA-seq, single-cell RNA sequencing; snRNA-seq, single-nucleus RNA sequencing; ST, spatial transcriptomics; TF, transcription factor; TFBS, transcription factor-binding sites; VAE, variational autoencoder; WSI, whole-slide images.
